# Supplementary material for: Copy number load predicts outcome of metastatic colorectal cancer patients receiving bevacizumab combination therapy
Source: Nat Commun. 2018 Oct 5;9:4112. doi: 10.1038/s41467-018-06567-6 (PMC6173768; doi:10.1038/s41467-018-06567-6)

Copy Number Load predicts Outcome of Metastatic Colorectal Cancer Patients receiving Bevacizumab combination therapy

Smeets et. al

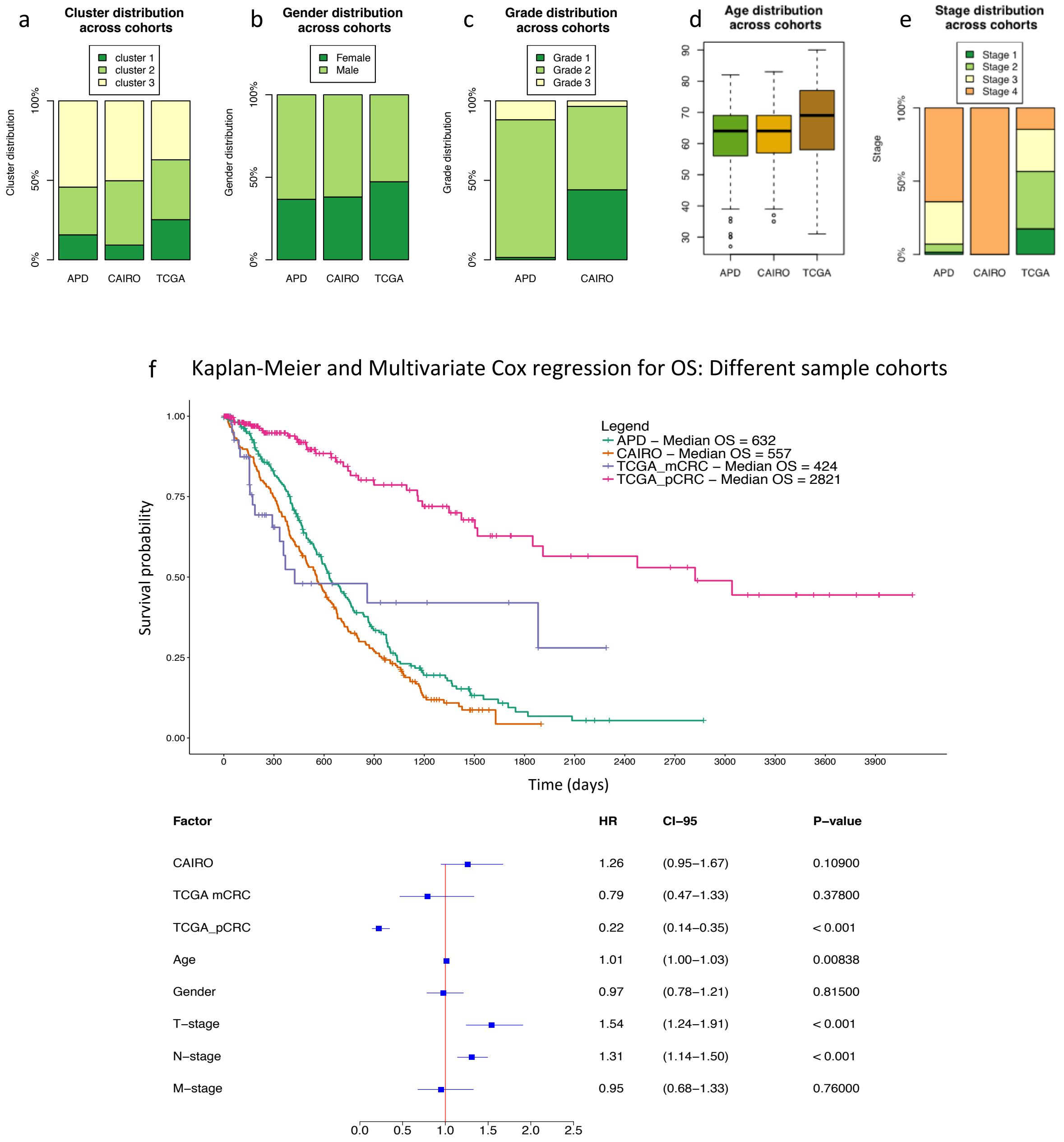

Supplementary Fig. 1 Distribution of clinical characteristics and overall survival among the different cohorts. **a-e** Distribution of (a) clusters, (b) gender, (c) grade, (d) age and (e) stage in the 3 different cohorts (APD, CAIRO and TCGA). P-values are respectively 1.49 10E-7 (for CNA cluster distribution), 0.011 (gender), <10E-16 (grade) and <10E-16 (stage) by Chi2-square. Age distribution per cohort was assessed by ANOVA (P=6.76 10E-8). **f** Overall survival analysis using Cox regression for the APD, CAIRO, TCGA metastatic samples (stage 4) and TCGA non-metastatic samples, while correcting for covariates (age, gender and TNM stage). APD was considered the reference cohort.

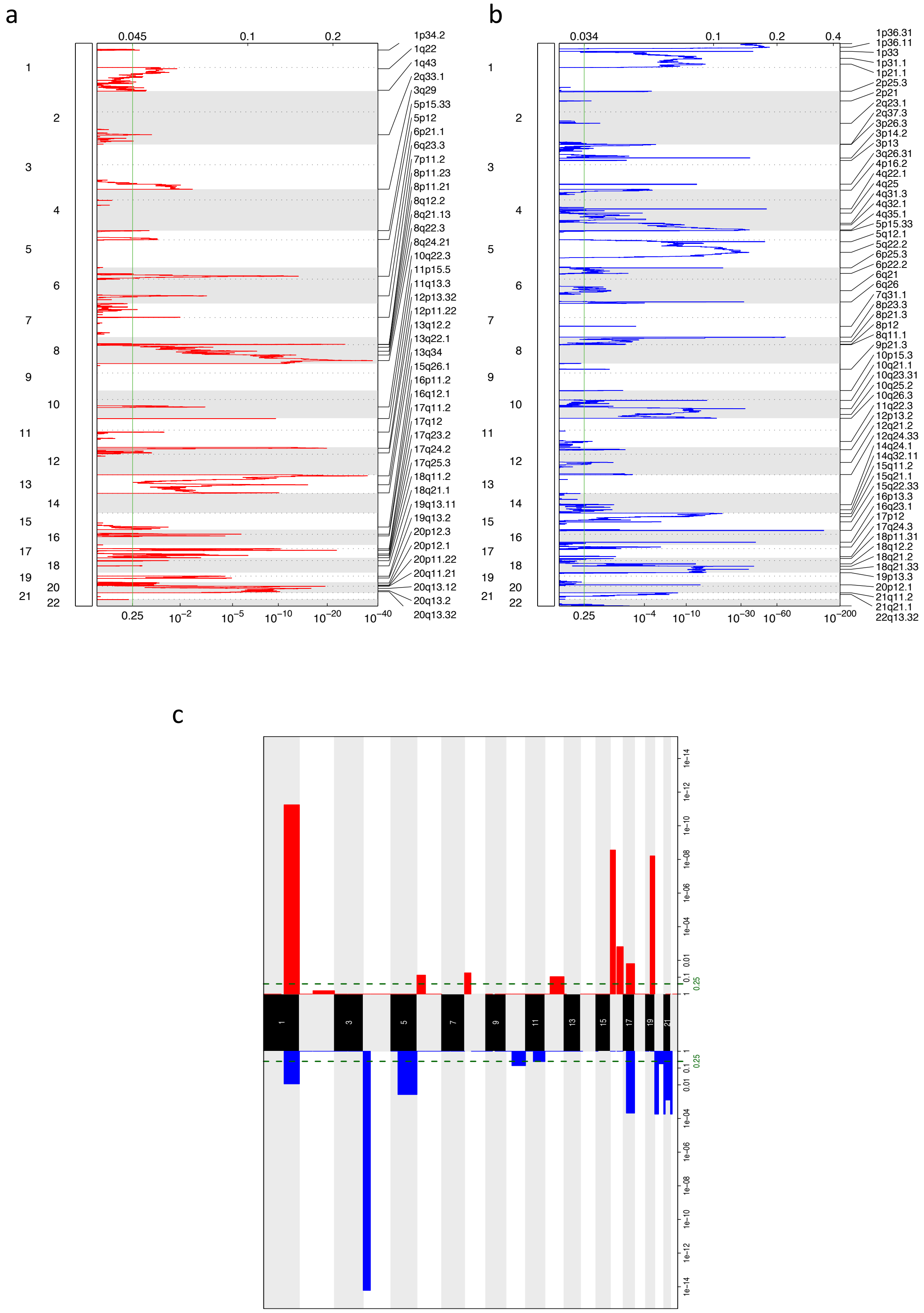

Supplementary Fig. 2. Recurrent CNAs in primary and metastatic colorectal cancer (n=908). **a, c** Recurrent amplifications (red) and deletions (blue) are represented. Focal amplifications are presented in (a), focal deletions in (b) and whole-arm amplifications and deletions in (c). The green lines represent the significance threshold at q<0.25. In total 43 recurrent focal amplifications and 59 focal deletions were identified.

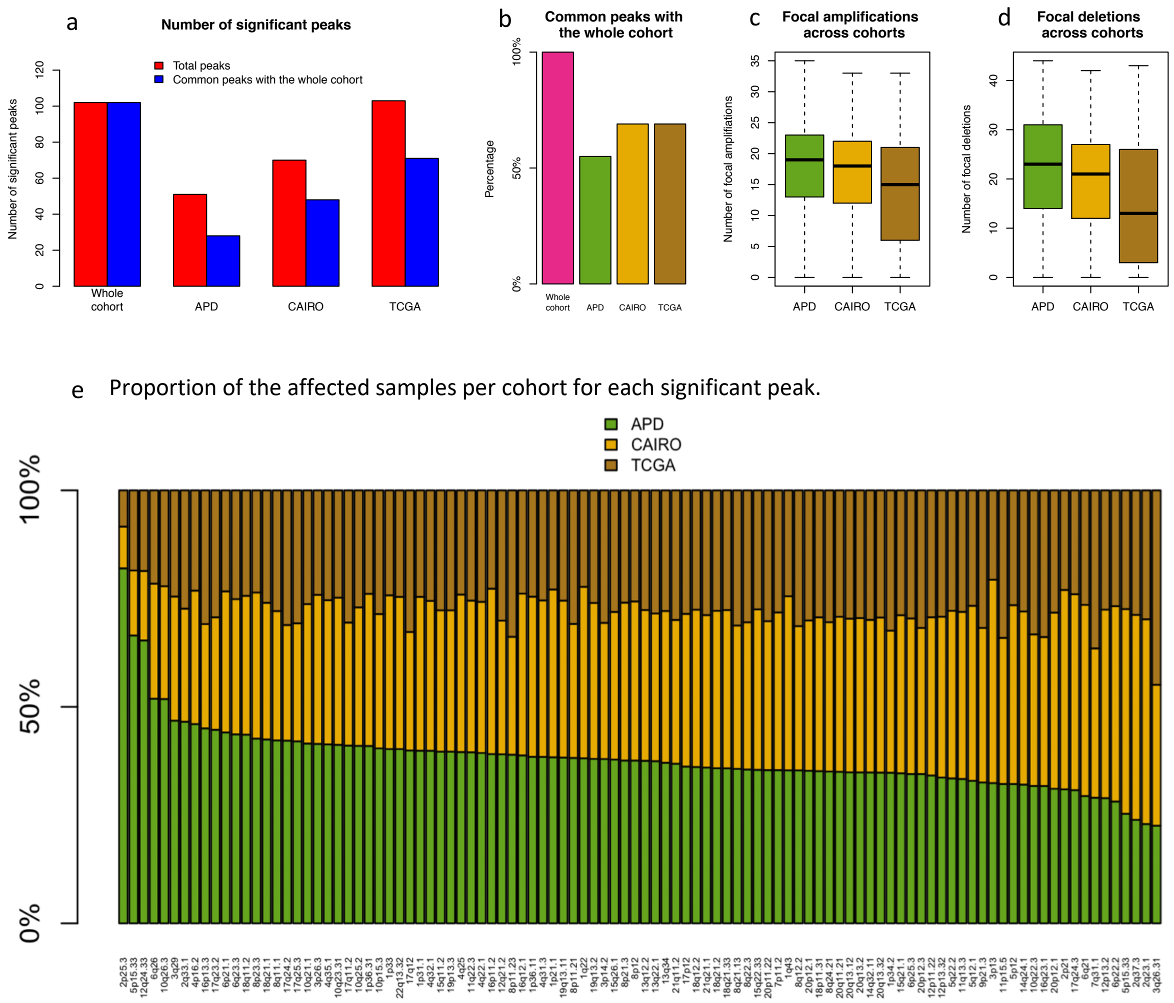

Supplementary Fig. 3. Recurrent CNAs presented per cohort. **a** The total number of significant GISTIC peaks detected in each cohort (whole cohort, APD, CAIRO and TCGA) is shown in red. For each cohort, we also calculated how many of these peaks were detected in the whole cohort (102 focal peaks) as shown in blue. **b** The proportion of peaks per cohort that are present in the whole cohort is shown (blue divided by red number of peaks in the plot a). **c, d** The average number of amplifications or deletions detected per samples across the 3 cohorts (APD, CAIRO and TCGA). Box plots show the median, the 25th and 75th percentiles, Tukey whiskers (median ± 1.5 times interquartile range) **e** Percentage of samples (%) with one of the 102 focal events stratified for the 3 cohorts (APD, green; CAIRO, yellow and TCGA, brown).

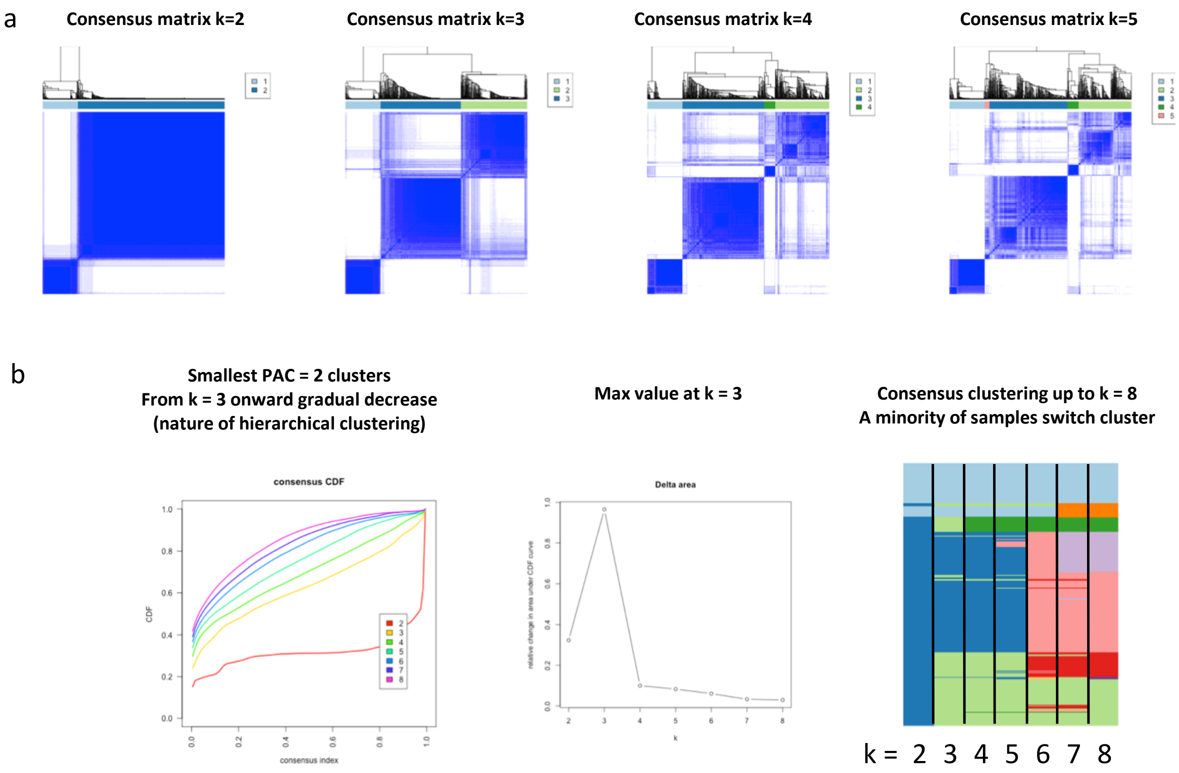

c Clustering reproducibility

	mCRC cluster 1	mCRC cluster 2	mCRC cluster 3
All CRC cluster 1	44	2	0
All CRC cluster 2	4	154	26
All CRC cluster 3	0	14	228

d Distribution of cohorts within the different clusters

	Cluster 1	Cluster 2	Cluster 3
APD	12%	31%	57%
CAIRO	9%	40%	50%
TCGA	25%	38%	37%

	Cluster 1	Cluster 2	Cluster 3
APD	13%	31%	56%
CAIRO	9%	40%	50%
TCGA_mCRC	6%	38%	56%
TCGA_pCRC	28%	39%	33%

Supplementary Fig. 4. Comparison of unsupervised hierarchical consensus clustering of copy number profiles of metastatic colorectal cancer (n=472) and total colorectal cancer (n=908) and selection of 3 subgroups. **a, b** Consensus matrix of samples clustered according to 2, 3, 4 or 5 subgroups. **c** When performing consensus clustering on mCRC or total CRC separately, 90.3% of the tumors are classified in the same cluster, indicating a high clustering reproducibility. **d** The samples from the 3 different cohorts (3 different technologies) are distributed similarly throughout the 3 clusters. The increase of samples allocated to cluster 1 for the TCGA samples can simply be explained by the fact that primary CRCs have on average more MSI (12-15%) compared to metastatic CRCs (3-5%). When splitting TCGA samples in primary and metastatic CRC this effect is clearly visible.

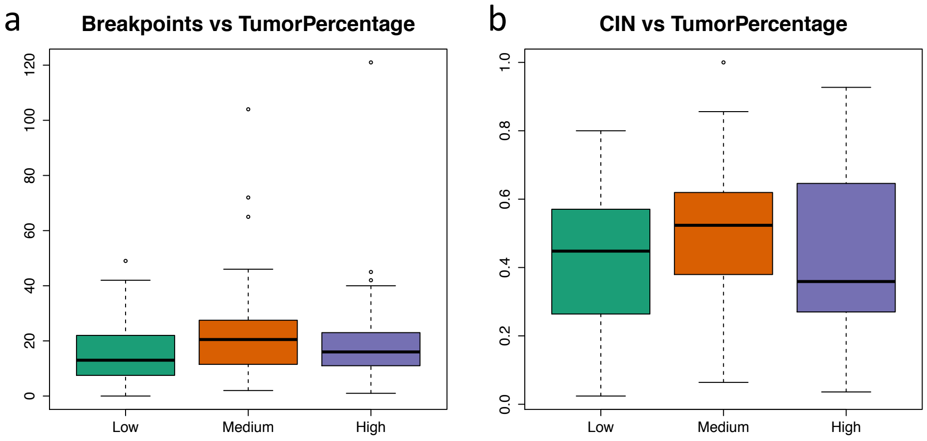

Supplementary Fig. 5. Effect of tumor percentage on chromosomal instability. **a** The number of CNA breakpoints detected in tumors with low (<40%), medium (40-60%) and high (>60%) tumor percentage are depicted. **b** Chromosomal instability (CIN) detected in tumors with a low (<40%) medium (40-60%) and high (>60%) tumor percentage are depicted. Box plots show the median, the 25th and 75th percentiles, Tukey whiskers (median ± 1.5 times interquartile range).

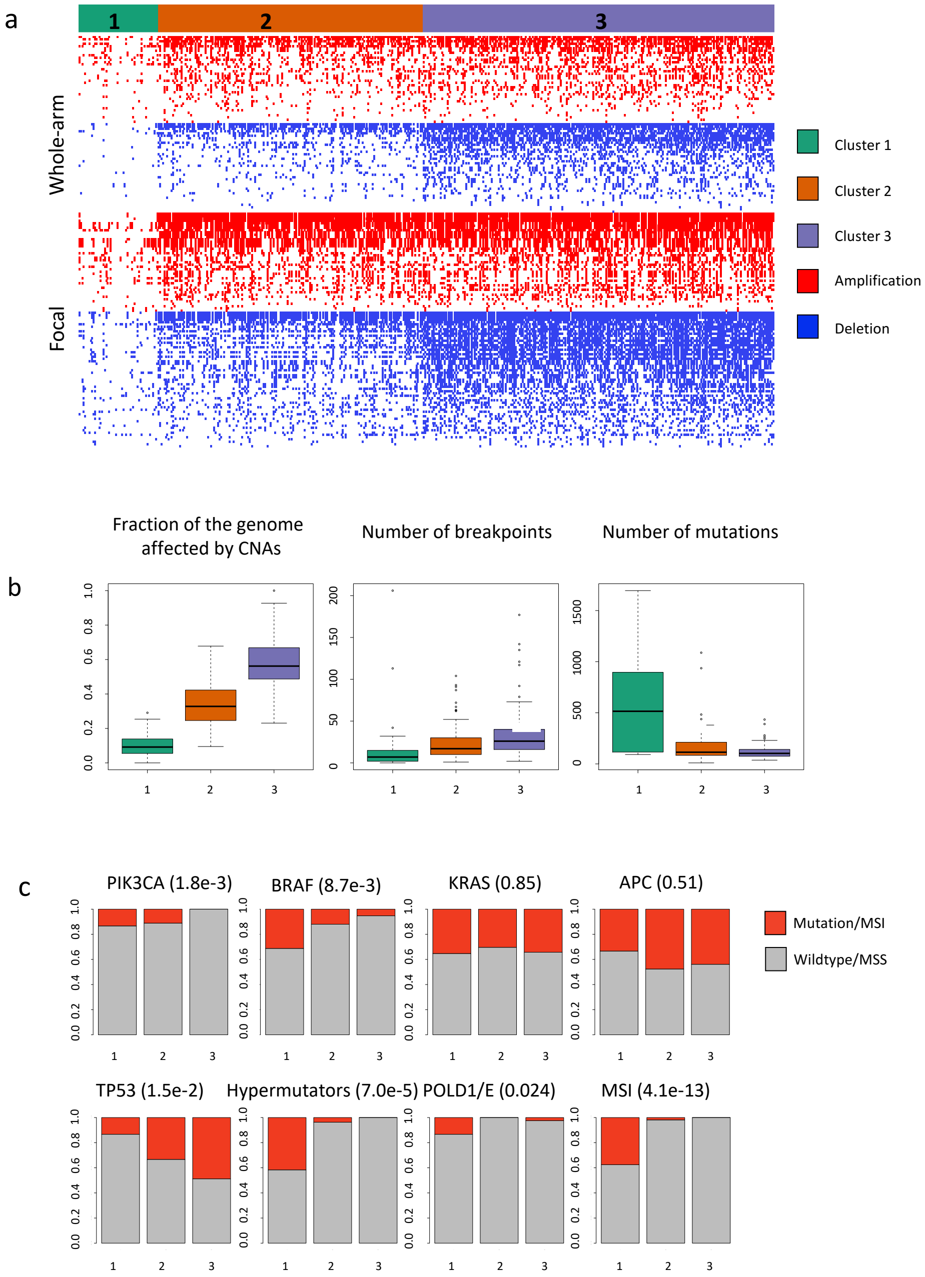

Supplementary Fig. 6. Unsupervised hierarchical Ward consensus clustering of copy number profiles of mCRC. **a** Unsupervised hierarchical clustering performed on the metastatic tumors only classified tumors into 3 consensus CNA subgroups termed clusters 1-3 based on recurrent CNAs as determined by GISTIC. Presence of recurrent amplifications (red) and deletions (blue) for each sample are represented. **b, c** Genomic characterization of the 3 clusters revealed that the characteristics of the clusters are almost identical to the clusters determined in primary and metastatic colorectal cancer combined. Cluster 1 was enriched for MSI tumors and hypermutators as well as tumors with mutations in BRAF and PIK3CA. In contrast, Clusters 2 and 3 were enriched for tumors with mutations in TP53, a high copy number instability and a higher number of chromosomal breakpoints. Mutations in KRAS and APC are found across all clusters. Box plots show the median, the 25th and 75th percentiles, Tukey whiskers (median ± 1.5 times interquartile range)

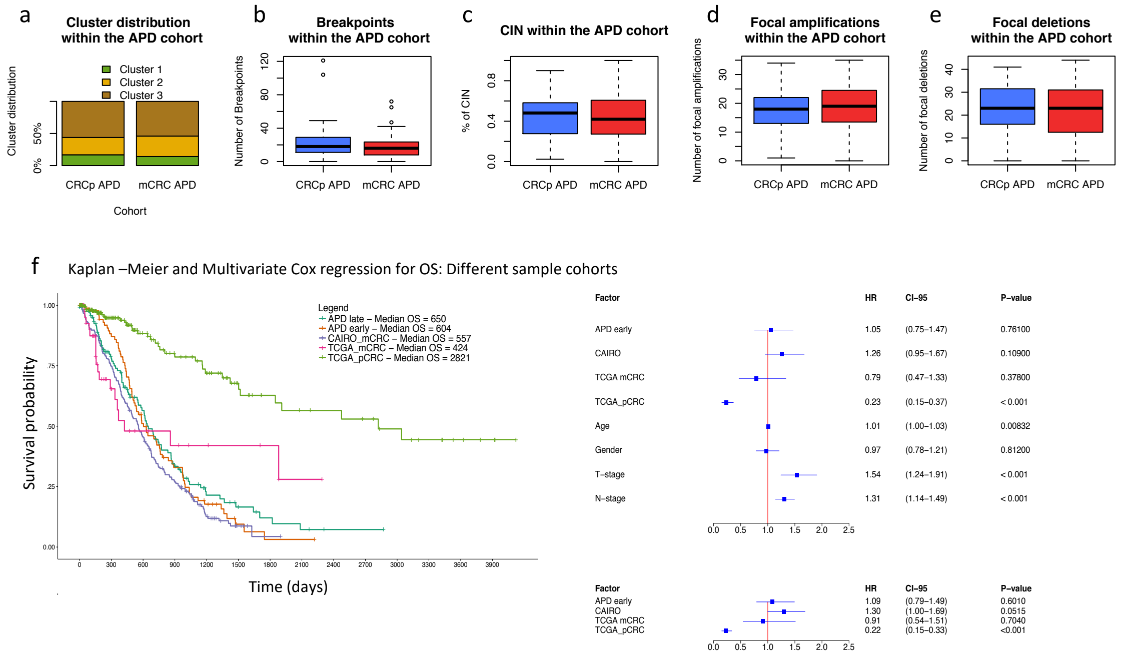

Factor	HR	CI–95	P–value
APD early	1.05	(0.75–1.47)	0.76100
CAIRO	1.26	(0.95–1.67)	0.10900
TCGA mCRC	0.79	(0.47–1.33)	0.37800
TCGA_pCRC	0.23	(0.15–0.37)	< 0.001
Age	1.01	(1.00–1.03)	0.00832
Gender	0.97	(0.78–1.21)	0.81200
T–stage	1.54	(1.24–1.91)	< 0.001
N–stage	1.31	(1.14–1.49)	< 0.001

Factor	HR	CI–95	P–value
APD early	1.09	(0.79–1.49)	0.6010
CAIRO	1.30	(1.00–1.69)	0.0515
TCGA mCRC	0.91	(0.54–1.51)	0.7040
TCGA_pCRC	0.22	(0.15–0.33)	<0.001

Supplementary Fig. 7. Characterization and comparison of early and late biopsies. **a** Distribution of the 3 CNA clusters in the APD tumors collected at resection (n=80, CRCp, APD early) and at metastasis (n=124, mCRC, APD-late), as well as **b** the number of breakpoints, **c** the average degree of chromosomal instability (CIN), **d** the number of focal amplifications and (e) focal deletions. For none of the comparisons a significant effect was observed (p-values > 0.05). Box plots show the median, the 25th and 75th percentiles, Tukey whiskers (median ± 1.5 times interquartile range). **f** OS analysis using Cox regression for the i) the 80 CRCp APD patients, ii) the 124 mCRC patients from APD, iii) the 205 mCRC patients from CAIRO, iv) the 63 mCRC patients from TCGA, and v) the 436 pCRC patients from TCGA while correcting for the relevant covariates (age, gender and TNM stage). The 402 mCRC patients were considered as reference cohort.

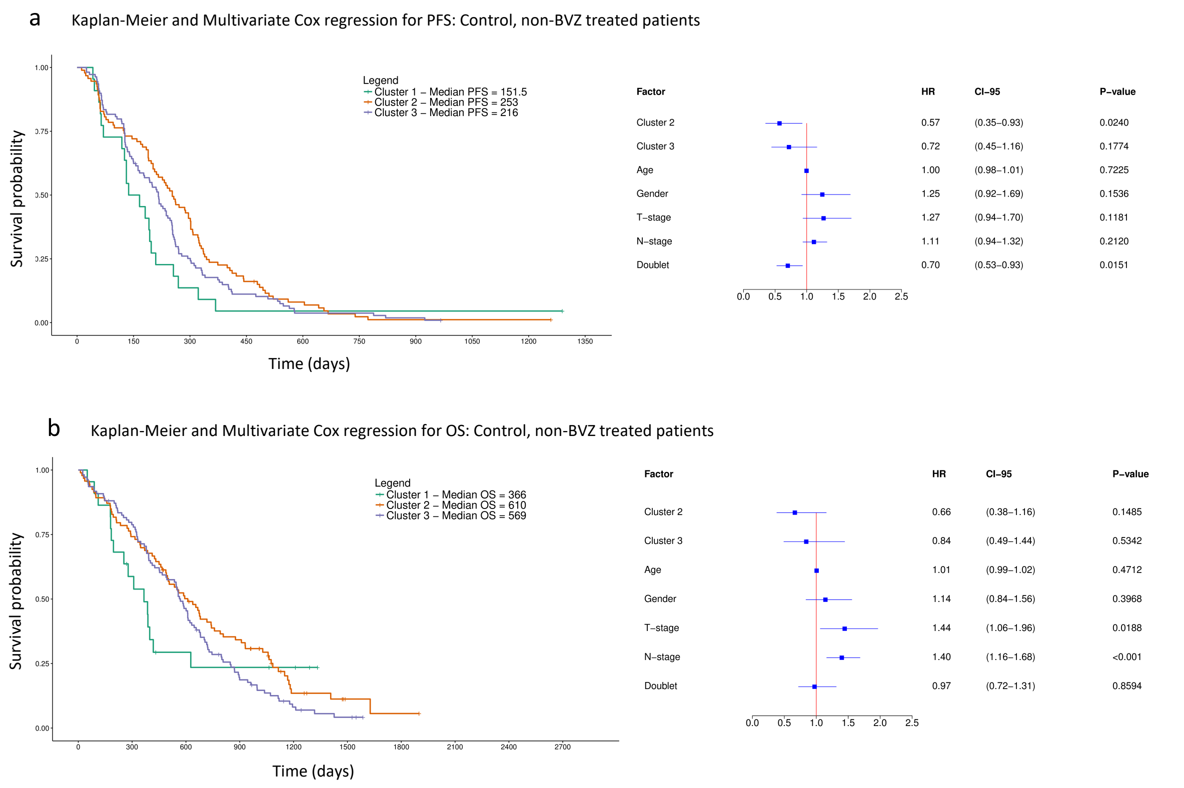

Supplementary Fig. 8. Kaplan Meier plots and multivariable Cox regression of the different clusters with OS and PFS in non-BVZ treated mCRC samples (n=224). **a** Kaplan Meier plots and multivariable Cox regression for progression free survival. **b** Kaplan Meier plots and multivariable Cox regression for overall survival.

a Kaplan-Meier and Multivariate Cox regression for PFS: BVZ treated patients Cluster 1

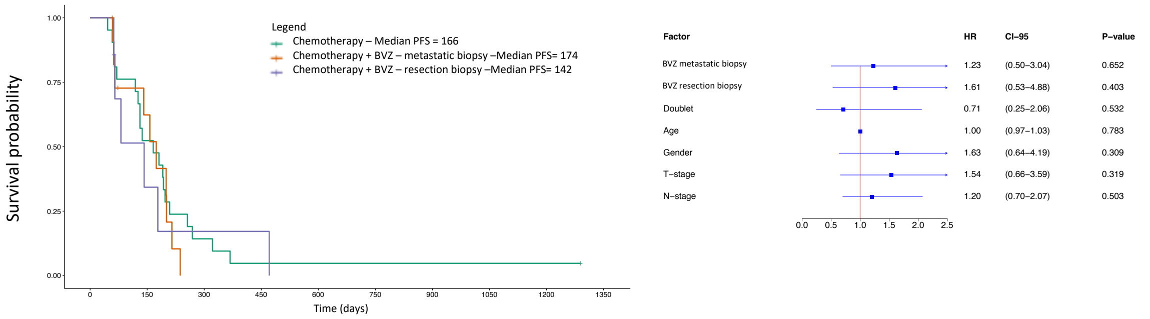

b Kaplan-meier and Multivariate Cox regression for PFS: BVZ treated patients Cluster 2

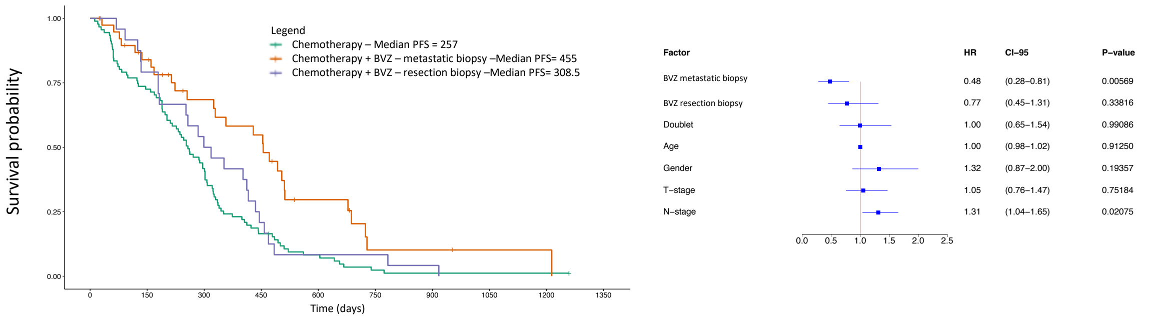

c Kaplan-Meier and Multivariate Cox regression for PFS: BVZ treated patients Cluster 3

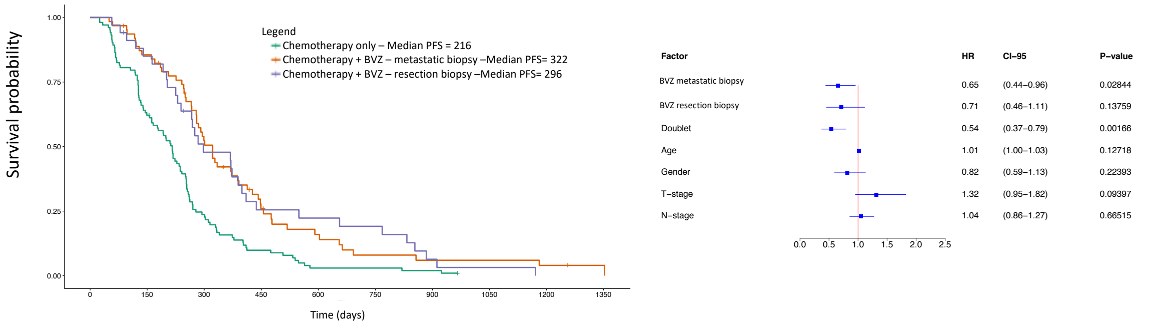

Supplementary Fig. 9. Cox regression survival analysis assessing the effect of BVZ ± chemotherapy in CRC patients belonging to CNA cluster (a) 1, (b) 2 or (c) 3, respectively. Standard-of-care chemotherapy was used as a reference, and the APD cohort receiving BVZ was stratified into CRCp APD patients (n=106) and mCRC APD patients (n=134). All analyses were corrected for chemotherapy backbone (doublet), age, T and N stage.

a Kaplan-Meier and Multivariate Cox regression for OS: BVZ treated patients Cluster 1

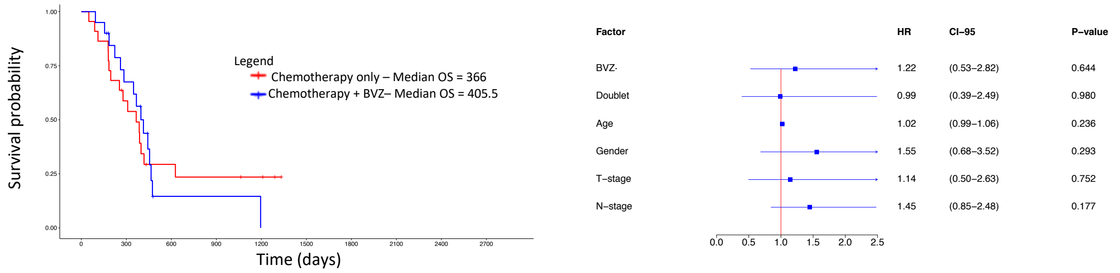

b Kaplan-Meier and Multivariate Cox regression for OS: BVZ treated patients Cluster 2

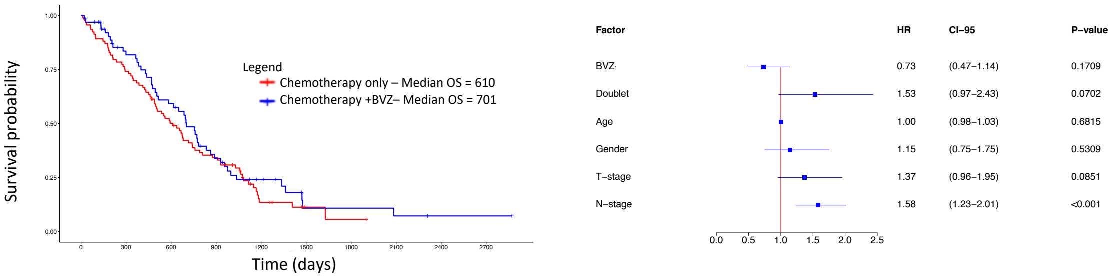

c Kaplan-Meier and Multivariate Cox regression for OS: BVZ treated patients Cluster 3

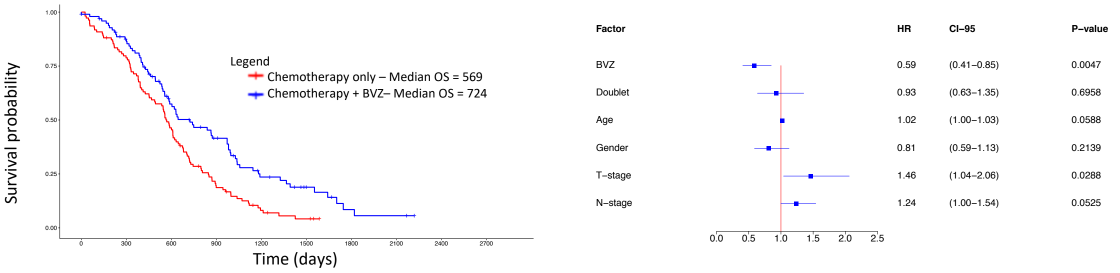

d Kaplan-Meier and Multivariate Cox regression for OS: BVZ treated patients Cluster 2 + 3

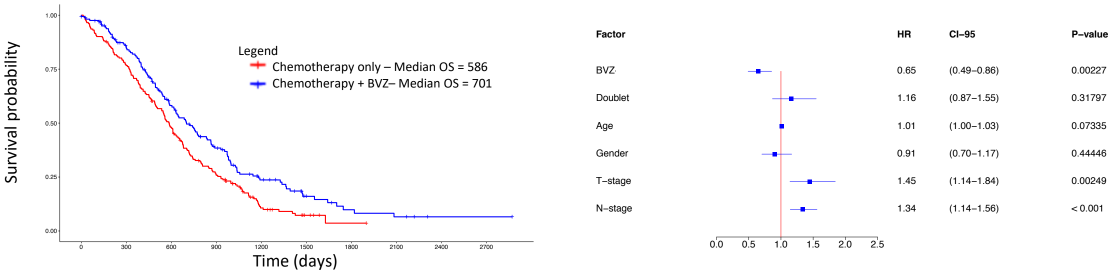

Supplementary Fig. 10. Comparison of patients treated with bevacizumab to those not treated with bevacizumab for each of the mCRC clusters and the effect on OS. **a,b** Patients from cluster 1 and 2 have no additional benefit for OS from bevacizumab treatment. **c** In contrast, patients from clusters 3 show additional benefit when treated with bevacizumab compared to patients not treated with bevacizumab. **d** Similar results were obtained when combining patients from clusters 2 and 3 in one group.

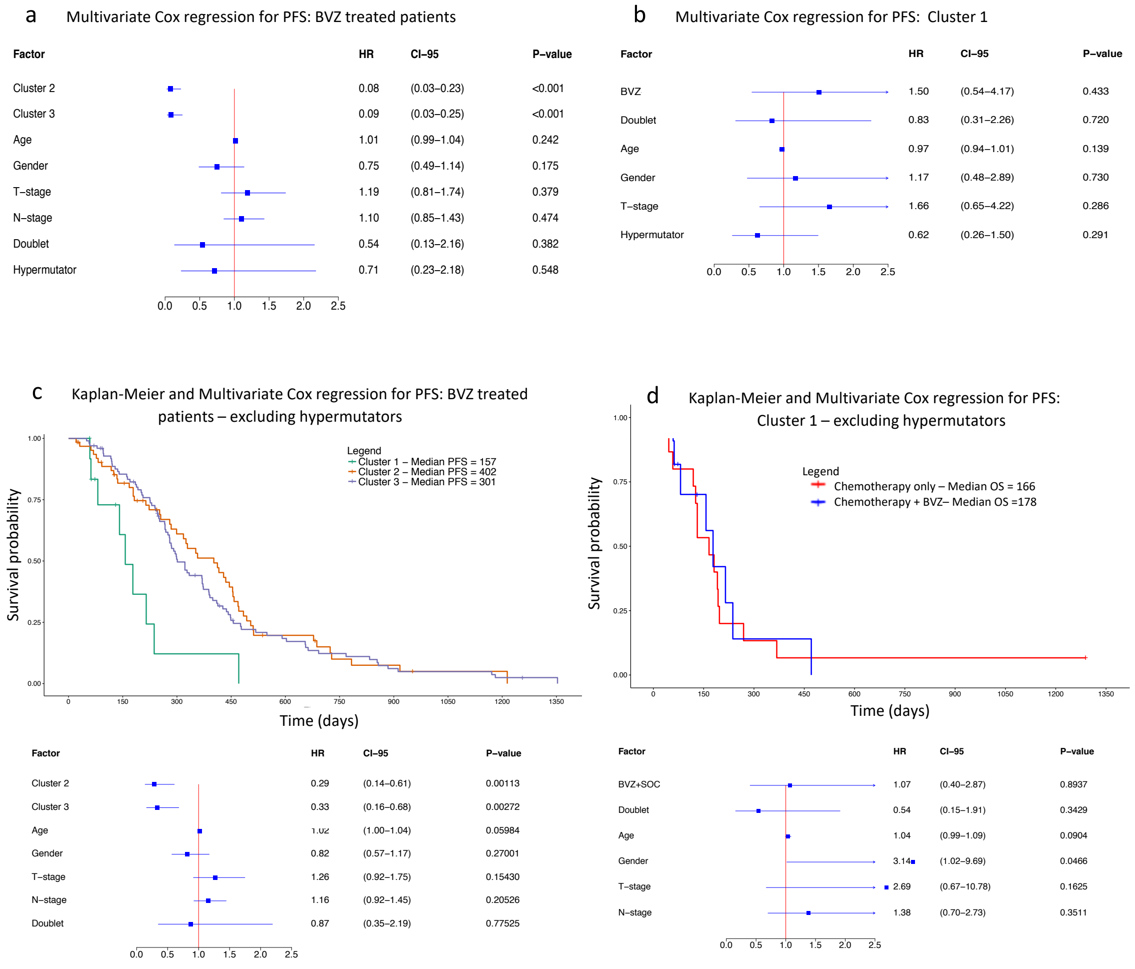

Supplementary Fig. 11. a Cox regression multivariate analysis testing survival differences between the 3 CNA clusters (while considering CNA cluster 1 as a reference) in BVZ-treated patients. This analysis was corrected for standard covariates, as well as hypermutator status. **b** Cox regression multi-variate analysis testing BVZ versus no BVZ treatment in CNA cluster 1 patients only, while including hypermutator status as one of the co-variates (in addition to chemotherapy backbone (doublet), age, gender, T-stage). **c** Cox regression survival analyses on BVZ-treated patients only, comparing the effects between the 3 CNA clusters (considering CNA cluster 1 as a reference), while excluding hypermutator samples. The analysis was corrected for age, gender, T and N stage and chemotherapy backbone (doublet). **d** Cox regression survival analyses comparing BVZ versus non-BVZ-treated patients in CNA cluster 1 patients (considering the non-BVZ treated arm as a reference), while excluding hypermutator samples. The analysis was corrected for age, gender, T and N stage and chemotherapy backbone (doublet).

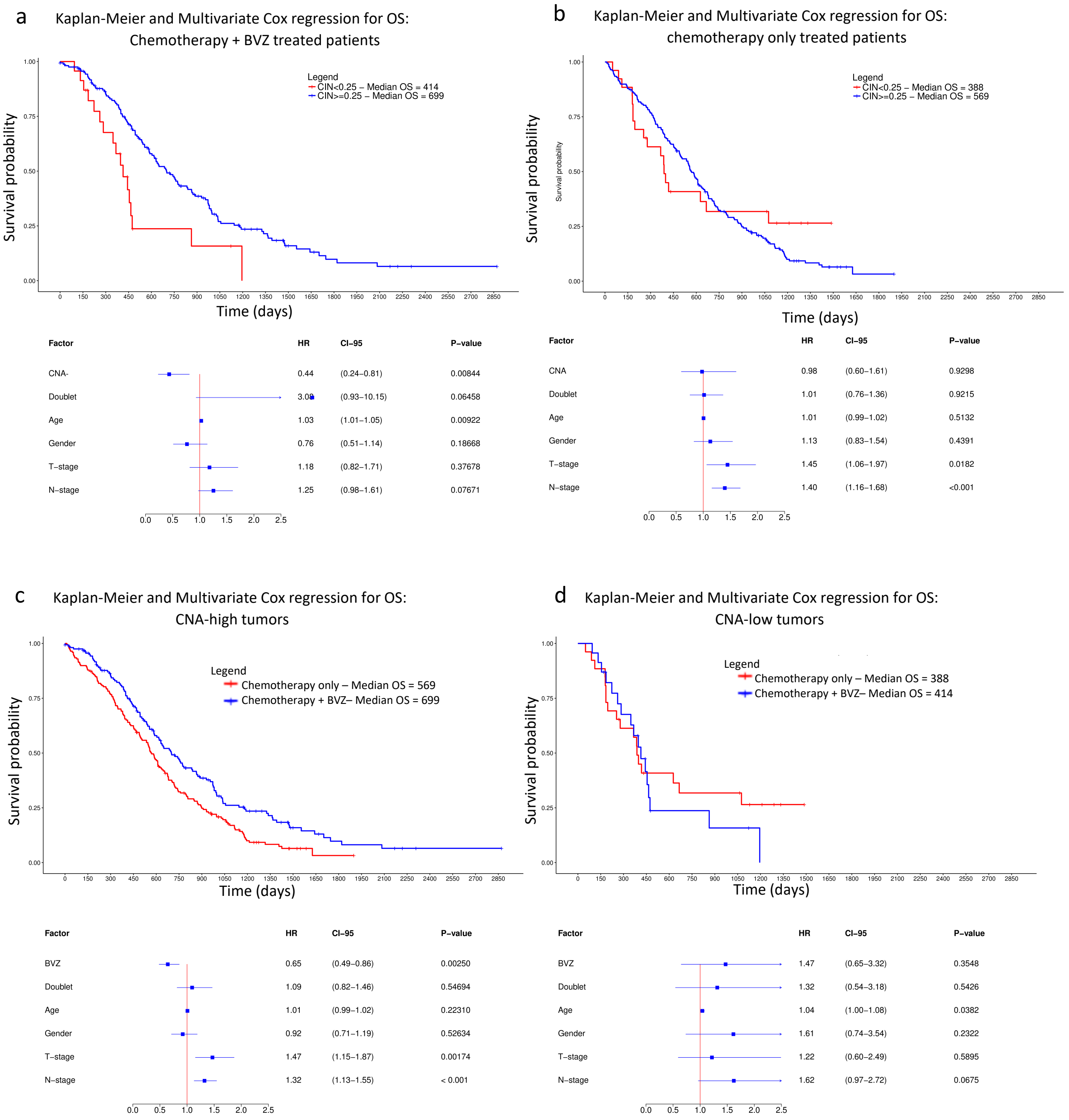

Supplementary Fig. 12. Comparison of CNA-high with CNA-low tumors and effect on OS. Patients were stratified in CNA-high and CNA-low tumors based on the number of CNAs. **a** For the bevacizumab treated tumors, a higher number of CNAs correlates with a longer progression-free survival and overall survival compared to tumors with a lower number of CNAs. **b** This effect is not present for tumors not treated with bevacizumab. **c** CNA-high patients that are treated with bevacizumab have a significantly better progression free survival and overall compared to CNA-high patients treated with standard-of-care chemotherapy. **d** This effect is not observed for CNA-low tumors.

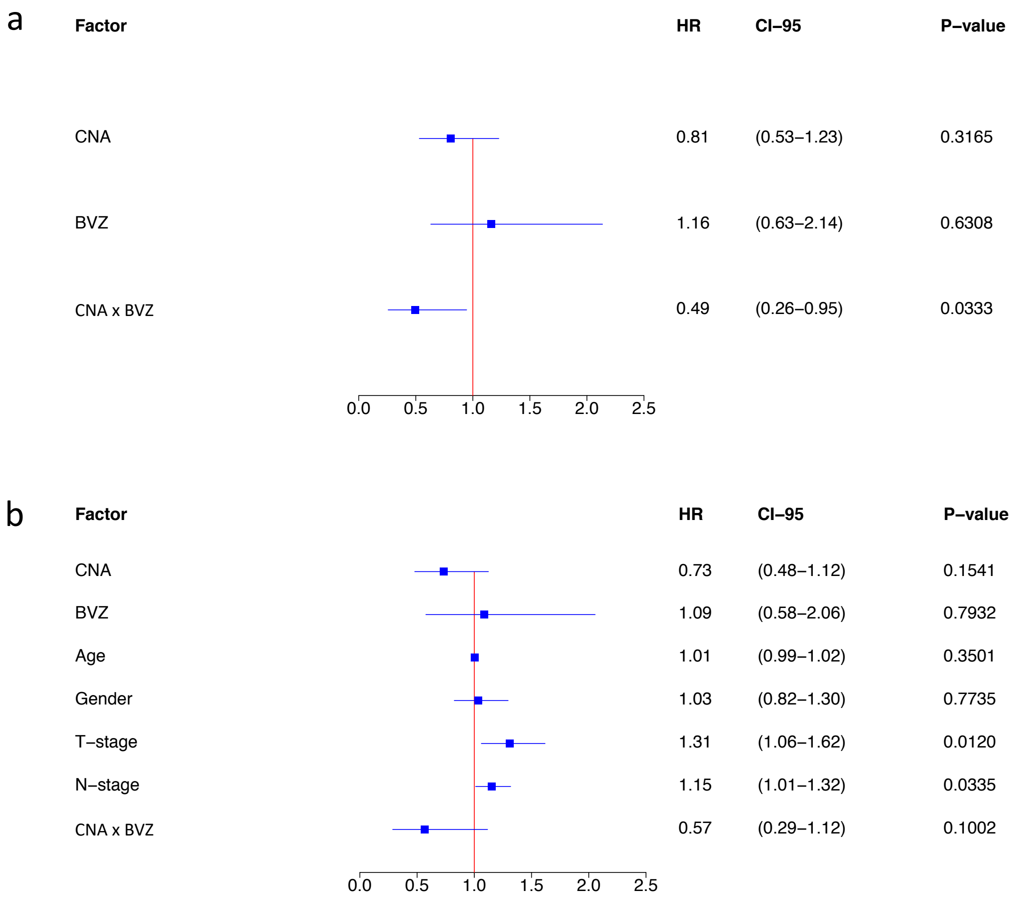

Supplementary Fig. 13. Interaction analysis on all BVZ and non-BVZ treated tumors using CIN and BVZ treatment combination as interaction variable. **a** Single variate analysis showing no effect of CIN and BVZ as individual explanatory variables. It is the interplay between CIN and BVZ treatment that predicts treatment outcome, with CIN-high tumors treated with BVZ showing an improved response. **b** Multivariate analysis using CIN and BVZ treatment as interaction variable.

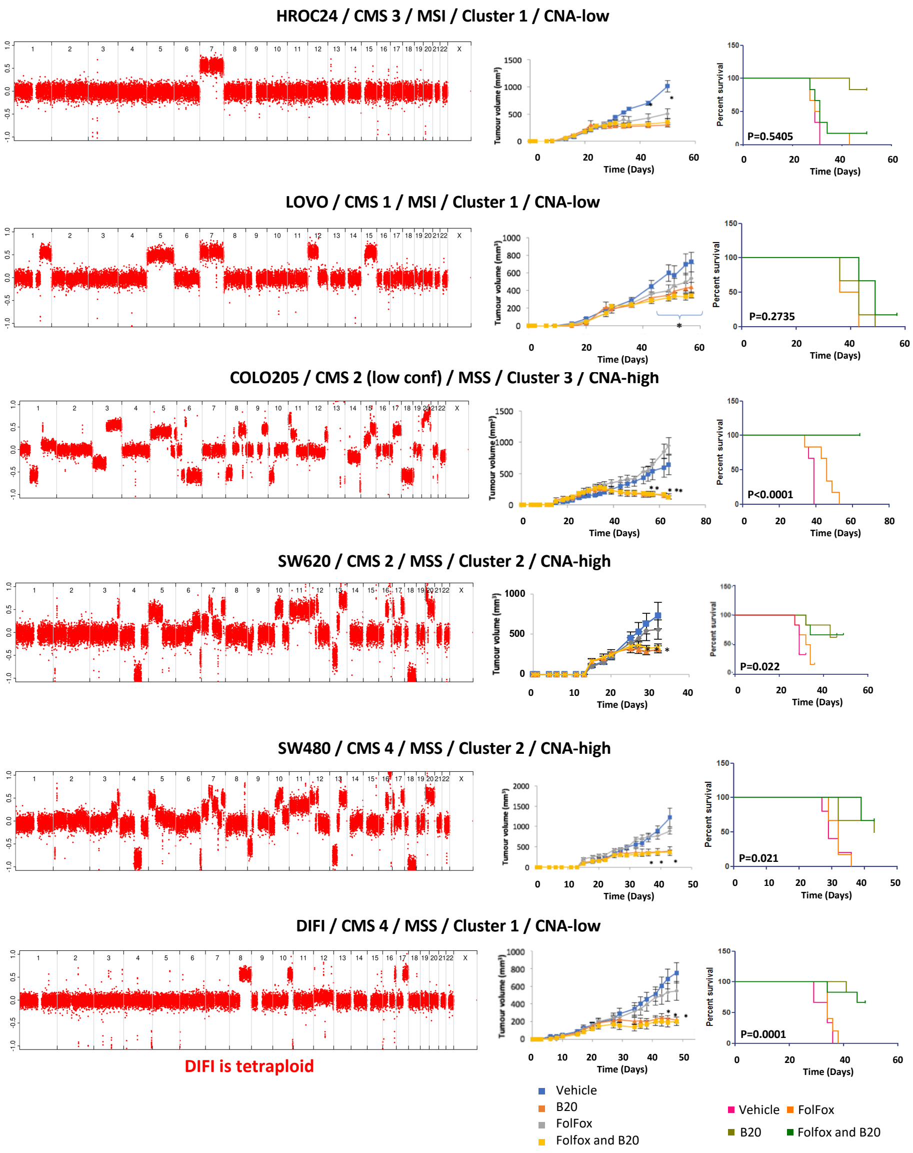

Supplementary Fig. 14. *In vivo* experiments and IHC analyses on xenografts. **a, g** Whole-genome copy number profile and growth curves of xenografts and analysis of the tumor sizes of the cell lines. Error bars represent s.e.m. of 6 animals per group. Student T test *=p< 0.05.

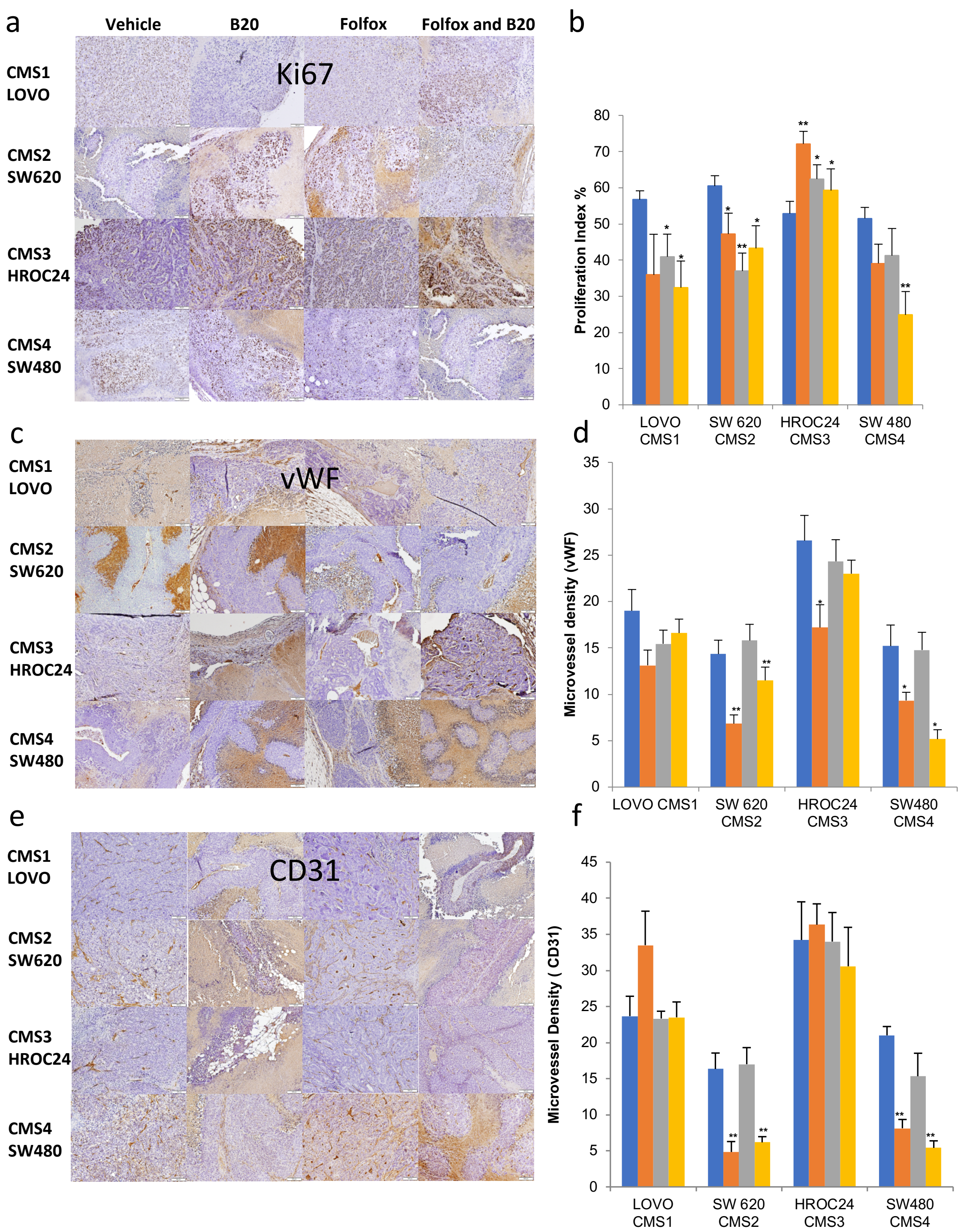

Supplementary Fig. 15. Immunohistochemical analysis of proliferation and microvessel density in CMS 1-4 subtyped cell line xenografts treated with FOLFOX and B20.

a Representative micrographs (100X) of CMS subtyped cell line xenografts stained with DAB probe for cell proliferation marker Ki67 (1:150 Rabbit α-KI67, heat mediated antigen unmasking) in 4 cell lines subtyped according to Guinney *et al* (CMS1: LOVO, CMS2: SW620, CMS3: HROC24, CMS4: SW480. Images were analysed by colour deconvolution in Image J and counting all positive brown nuclei. **b** Analysis of the CMS subtyped cell line xenografts stained with DAB probe for cell proliferation marker Ki67. 3 xenografts per cell line were analysed with 4 images per xenograft. A significant reduction in proliferation is noted in both CMS 2 and CMS 4 xenografts treated with

Folfox and B20 compared to Folfox alone. **c** Representative micrographs (100X) of CMS subtyped cell line xenografts stained with DAB probe for blood vessel marker von Willibrand Factor (vWF) (1:75 Rabbit α-mouse vWF Abcam, heat mediated antigen unmasking) in cell lines subtyped according to Guinney *et al*. **d** Analysis of the CMS subtyped cell line xenografts stained with DAB probe for blood vessel marker vWF. Images were analysed by applying a 15000 $pixel^2$ grid over the image in Image J and counting the number of times positive vessels cross the grid. 3 xenografts per cell line were analysed with a minimum of 4 images per xenograft. A significant reduction ($p<0.05$) in the number of vWB positive vessels in CMS 2 and 4 xenografts treated with Folfox followed by B20 compared to Folfox alone, however this effect is not noted in CMS1 or 3 xenografts. **e** Representative micrographs (100X) of CMS subtyped cell line xenografts stained with DAB probe for tumour blood vessel marker CD31 (1:25 Rabbit α-mouse CD31 Santa Cruz, heat mediated antigen unmasking) in cell lines subtyped according to Guinney *et al*. **f** Analysis of the CMS subtyped cell line xenografts stained with DAB probe for tumour blood vessel marker CD31. Images were analysed by applying a 15000 $pixel^2$ grid over the image in Image J and counting the number of times positive vessels cross the grid. 3 xenografts per cell line were analysed with a minimum of 4 images per xenograft. A highly significant reduction ($p<0.005$) in the number of CD31 positive vessels in CMS 2 and 4 xenografts treated with Folfox followed by B20 compared to Folfox alone, however this effect is not noted in CMS1 or 3 xenografts Error bars represent standard error of the mean (SEM) *= $p<0.05$ **=$P<0.005$.

Supplementary Table 1. Expanded clinical information of the different cohorts.

		APD		MOMA		CAIRO		TCGA		TOTAL	
		N=21	%	N=8	%	N=20	%	N=49	%	N=100	%
**Gender**											
	**Female**	79	36.	31	38.3	78	38.0	30	6.0	218	21.
	**Male**	134	62.	50	61.7	127	62.0	33	6.6	344	34.
	**Missing values**	2	0.9	0	0.0	0	0.0	436	87.	438	43.
**Age, years**											
	**>65**	87	40.	21	25.9	96	46.8	33	6.6	237	23.
	**≤65**	123	57.	60	74.1	109	53.2	30	6.0	322	32.
	**Missing values**	5	2.3	0	0.0	0	0.0	436	87.	441	44.
**T-classification**											
	**1**	2	0.9	1	1.2	0	0.0	0	0.0	3	0.3
	**2**	17	7.9	3	3.7	12	5.9	0	0.0	32	3.2
	**3**	138	64.	29	35.8	144	70.2	40	8.0	351	35.
	**4**	50	23.	15	18.5	44	21.5	23	4.6	132	13.
	**Missing values**	8	3.7	33	40.7	5	2.4	436	87.	482	48.
**N-classification**											
	**0**	51	23.	8	9.9	62	30.2	8	1.6	129	12.
	**1**	79	36.	17	21.0	66	32.2	25	5.0	187	18.
	**2**	70	32.	22	27.2	66	32.2	30	6.0	188	18.
	**Missing values**	15	7.0	34	42.0	11	5.4	436	87.	496	49.
**M-classification**											
	**0**	88	40.	0	0.0	0	0.0	0	0.0	88	8.8
	**1**	122	56.	81	100.	205	100.	63	12.	471	47.
	**Missing values**	5	2.3	0	0.0	0	0.0	436	87.	441	44.
**MSI**											
	**Instable**	8	3.7	0	0.0	6	2.9	1	0.2	15	1.5
	**Stable**	121	56.	0	0.0	199	97.1	55	11.	375	37.
	**Missing values**	86	40.	81	100.	0	0.0	443	88.	610	61.
**KRAS**											
	**wt**	105	48.	33	40.7	0	0.0	30	6.0	168	16.
	**mut**	50	23.	46	56.8	0	0.0	16	3.2	112	11.
	**Missing values**	60	27.	2	2.5	205	100.	453	90.	720	72.
**BRAF**											
	**wt**	122	56.	**72**	88.9	0	0.0	45	9.0	239	23.
	**mut**	16	7.4	**7**	8.6	0	0.0	3	0.6	26	2.6
	**Missing values**	77	35.	**2**	2.5	205	100.	451	90.	735	73.
**Tumor location**											
	**colon**	157	73.	57	70.4	0	0.0	61	12.	275	27.
	**rectum**	56	26.	24	29.6	0	0.0	0	0.0	80	8.0
	**Missing values**	2	0.9	0	0.0	205	100.	438	87.	645	64.
**Therapy**											
	**BVZ**	2	0.9	0	0.0	0	0.0	0.0	0.0	2	0.2
	**FP-BVZ**	12	5.6	0	0.0	0	0.0	0.0	0.0	12	1.2
	**FP-OX-BVZ**	138	64.	0	0.0	0	0.0	0.0	0.0	138	13.
	**FP-IRI-BVZ**	37	17.	0	0.0	0	0.0	0.0	0.0	37	3.7
	**IRI-BVZ**	3	1.4	0	0.0	0	0.0	0.0	0.0	3	0.3
	**FP-OX-BVZ-mit**	2	0.9	0	0.0	0	0.0	0.0	0.0	2	0.2
	**FP-OX-BVZ-ima**	1	0.5	0	0.0	0	0.0	0.0	0.0	1	0.1
	**FP-OX-IRI-BVZ**	0	0.0	44	54.3	0	0.0	0.0	0.0	44	4.4
	**FP-OX-IRI-BVZ-mCT**	0	0.0	37	45.7	0	0.0	0.0	0.0	37	3.7
	**FP**	1	0.5	0	0.0	101	49.3	0.0	0.0	102	10.
	**FP-OX**	16	7.4	0	0.0	0	0.0	0.0	0.0	16	1.6
	**FP-IRI**	2	0.9	0	0.0	104	50.7	0.0	0.0	106	10.
	**FP-OX-cetuximab**	1	0.5	0	0.0	0	0.0	0.0	0.0	1	0.1
	**Missing values**	0	0.0	0	0.0	0	0.0	499	100	499	49.
**Total**		215	100	81	100	205	100	499	100	1000	100

wt=wild-type, mut=mutated, BVZ=bevacizumab, FP=fluoropyrimidin, IRI=irinotecan, OX=oxaliplatin, mit=mitomycin, ima=imatinib, mCT=metronomic chemotherapy, cet=cetuximab

Supplementary Table 2. Multivariate and univariate cox-regression within each of the clusters while stratifying patients for BVZ therapy and excluding patients not receiving BVZ in 2nd, 3d, 4th or 5th line.

	Factor	HR	95% CI lower	95% CI upper	P-Value
**Cluster 1 - PFS**	BVZ+SOC	1.112	0.521	2.374	0.784
	Doublet	0.973	0.363	2.606	0.956
	Age	1.007	0.978	1.037	0.633
	Gender	1.486	0.629	3.508	0.366
	T-stage	1.497	0.632	3.545	0.359
	N-stage	1.168	0.688	1.983	0.566
**Cluster 2 - PFS**	BVZ+SOC	0.616	0.392	0.968	0.0357
	Doublet	0.976	0.630	1.512	0.9138
	Age	0.995	0.973	1.018	0.6615
	Gender	1.417	0.930	2.160	0.1051
	T-stage	1.130	0.804	1.588	0.4812
	N-stage	1.240	0.980	1.569	0.0731
**Cluster 3 - PFS**	BVZ+SOC	0.688	0.488	0.969	0.03243
	Doublet	0.542	0.367	0.801	0.00211
	Age	1.009	0.993	1.026	0.25199
	Gender	0.826	0.600	1.139	0.24399
	T-stage	1.353	0.978	1.874	0.06819
	N-stage	1.057	0.865	1.291	0.58883

Supplementary Table 3. Report on the random forest classifier

Random forest classification Accuracy: 0.9246; 95% CI: (0.8976, 0.9464)			
**Statistics by class**	**Cluster 1**	**Cluster 2**	**Cluster 3**
**Sensitivity**	0.9286	0.861	0.9718
**Specificity**	0.9954	0.9638	0.9012
**Balanced Accuracy**	0.962	0.9124	0.9365

Supplementary Note 1. Workflow of the samples profiled using low-coverage whole-genome and whole-exome sequencing

After estimating tumor content by histopathology, tumor DNA was extracted and subjected to stringent DNA quality criteria prior to DNA library preparation (>0.5µg total yield and concentration > 7.5 ng/µl as determined by picogreen). After quantification with qPCR, respectively 238 and 93 DNA libraries for the AngioPredict and MoMa cohort were sequenced on a HiSeq2500 (Illumina) generating 1x50bp reads at low coverage (±0.1x). Only samples with more than 1 million mapped reads and a mean absolute pair-wise deviation lower than 0.4 were used in further analyses. Furthermore, we performed a manual check on each of the profiles to ensure sufficiently high tumor content resulting in 215 and 81 CNA profiles for the AngioPredict and MoMa cohort respectively. Whole-exome enrichment was performed on samples for which > 250ng of DNA library was available and passed the qualtity check for CNA profiling. The resulting libraries were sequenced on a HiSeq2500 generating 2x100bp reads. Only samples with a coverage > 10x and more than 50% of the exome covered by more than 10x were used in further analyses.

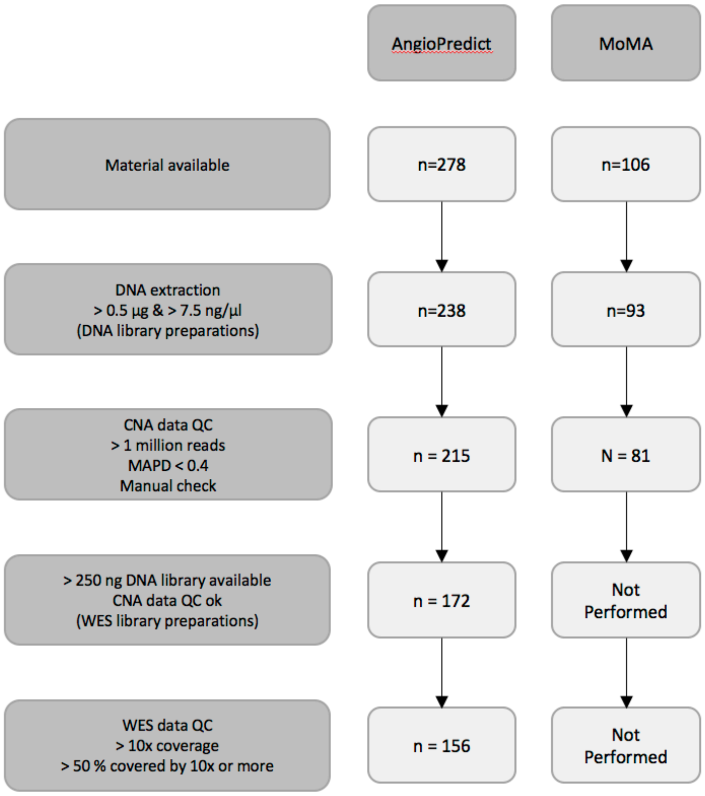

Supplement: Supplementary file 1 — Supplementary Information [file 41467_2018_6567_MOESM1_ESM.pdf]
